# The contribution of phenolic endocrine-disrupting chemicals to breast cancer risk: A comprehensive bioinformatics analysis

**DOI:** 10.1038/s41598-026-39706-x

**Published:** 2026-02-11

**Authors:** Yanhong Dou, Xiongxiong Li, Meng Li, Jin Shang, Ting Xu

**Affiliations:** Department of Breast Surgery, Xi’an People’s Hospital (Xi’an No. 4 Hospital), Xi’an, China

**Keywords:** Phenolic endocrine-disrupting chemicals, Network toxicology, Machine learning algorithm, Immune infiltration, Molecular docking, Molecular dynamics simulation, Breast cancer, Cancer, Computational biology and bioinformatics

## Abstract

Bisphenol A (BPA), nonylphenol (NP), and octylphenol (OP) are common environmental phenolic endocrine disruptors and widely used industrial chemicals that have garnered significant attention due to their potential to disrupt endocrine functions. These compounds are known to interfere with hormonal activities, particularly those related to estrogen, and are linked to the onset and progression of breast cancer. This study aims to systematically investigate the potential relationship between BPA, NP, and OP and breast cancer risk, along with their underlying molecular mechanisms, by synthesizing data from multiple databases. We initially acquired the chemical structures and SMILES representations of BPA, NP, and OP from the PubChem database. Subsequently, we utilized multiple databases, including the Comparative Toxicogenomics Database (CTD), SEA, and Swiss Target Prediction, t0 estimate their probable biological targets. The predicted targets were standardized and consolidated to form a comprehensive target database. Breast cancer-related targets were subsequently identified from the GeneCards and DisGeNET databases, and their overlap with the targets of BPA, NP, and OP was analyzed to pinpoint potential breast cancer risk targets. To elucidate the functional pathways involved, we conducted Gene Ontology (GO) and Kyoto Encyclopedia of Genes and Genomes (KEGG) enrichment analyses using the DAVID database. This analysis offered insights into the molecular pathways influenced by BPA, NP, and OP in the context of breast cancer. Additionally, we utilized machine learning algorithms, specifically Least Absolute Shrinkage and Selection Operator (LASSO) regression and Support Vector Machine (SVM), to identify nuclear targets linked to BPA, NP, and OP-induced breast cancer. These nuclear targets were further validated through differential expression analysis and Receiver Operating Characteristic (ROC) curve analysis using the GEO dataset GSE42568. We also performed a Single Gene Gene Set Enrichment Analysis (GSEA) to investigate the potential regulatory mechanisms of these nuclear genes in breast cancer. The infiltration of immune cells in breast cancer tissues was analyzed using single-sample gene set enrichment analysis (ssGSEA), and the correlation between nuclear targets and immune cell infiltration was examined. Finally, molecular docking and molecular dynamics simulations were conducted to assess the binding affinity and stability of BPA, NP, and OP with their nuclear targets. In this study, we integrated network toxicology, machine learning and multi-omics validation, and identified for the first time that BPA, NP and OP may induce breast cancer through 156 common targets; among them, MAOA, MGLL, ADRA2A, RPN2, GF1R and CTSD were identified as the key causative genes, with a diagnostic efficacy of 0.80–0.94 AUC. Mechanistically, these genes are concentrated in the GPCR/MAPK/JNK, sphingolipid, and prolactin signaling pathways, which regulate the Wnt/TGF-β/chemokine network and dramatically modify the immunological infiltration of nine classes of M0-M2 macrophages and CD4⁺ T cells. Molecular docking and kinetic simulations suggested the strong affinity of BPA for MGLL, and the complex was stabilized with ≥ 3 hydrogen bonds. In conclusion, phenolic endocrine disruptors may cause breast cancer through the “multi-target-immune microenvironment-metabolic reprogramming” axis, and MAOA, MGLL, ADRA2A, and RPN2 may serve as new targets for early detection and management.

**Supplementary Information:**

The online version contains supplementary material available at 10.1038/s41598-026-39706-x.

## Introduction

Endocrine-disrupting chemicals (EDCs) represent a newly identified category of environmental pollutants with endocrine activity, prevalent across the globe^1^ these substances are commonly detected in soil and aquatic systems. The high toxicity and bioaccumulation potential of EDCs, even at low concentrations, pose significant risks to aquatic environments, human health, and ecosystems^2^ studies have documented the presence of measurable levels of EDCs in the air of residential, office, and plastic manufacturing settings^3^ EDCs primarily enter the human body through dietary exposure, dermal contact, and inhalation^4^ dietary exposure is the predominant route, encompassing the consumption of EDC-contaminated seafood, food stored in plastic containers, and drinking water that is contaminated^5^ skin exposure ranks as the second most common route of absorption, occurring through direct contact with thermal paper, medical devices, and toys^5^ Inhalation of gases, mists, or dust containing EDCs constitutes a third significant exposure pathway. Numerous studies have established that EDCs share structural and functional similarities with certain hormones in the human body, which can lead to downstream gene activation and trigger intracellular signaling cascades by binding to estrogen receptors in various tissues 6 thereby impacting reproductive health and increasing the risk of hormone-dependent cancers^7^ Growing evidence indicates that the estrogenic properties of EDCs may correlate with the incidence of various cancers, including breast, prostate, and thyroid cancers^8–10^ Phenolic endocrine-disrupting chemicals (Phenolic EDCs) are a class of chemical substances characterized by their phenolic structure, with typical representatives including BPA, NP, and OP. Due to their structural stability and widespread use, they have become some of the most commonly detected endocrine disruptors in the environment and in human bodies^11^ which are extensively utilized in the production of phenolic compounds for consumer products such as surfactants, detergents, lubricants, plastics, food containers, and paper products^12,13^ With the ongoing increase in global production and consumption, a growing quantity of Phenolic EDCs is being released into the environment^14,15^ BC ranks as one of the most frequently diagnosed cancers, with an estimated 2.3 million new cases worldwide^16^. It is the fifth largest cause of cancer-related mortality^17^ Given that breast cancer is likely impacted by endocrine variables, it raises the question of whether Phenolic EDCs are associated to its development. Although this theory has been examined through observational studies, clear proof remains scarce. Therefore, by integrating network toxicology, machine learning, multi-omics immune infiltration and molecular modeling technologies, we will systematically elucidate the common molecular mechanisms and key targets of environmental endocrine disruptors BPA, NP, and OP affecting breast cancer, and lay the theoretical and data foundation for precise early warning and targeted prevention of environmentally related breast cancer.

BPA is considered a synthetic estrogen, and its estrogenic properties have been reported in in vivo studies^18^. Therefore, its primary mechanism of promoting breast cancer carcinogenesis can be attributed to its estrogenic activity, potentially inducing cell proliferation through the activation of estrogen receptors (ER). Nonylphenol likely exerts estrogen-like transcriptional activity on estrogen receptor-positive breast cancer cells by binding to Erα^19^. Additionally, studies have implyed that OP stimulates the growth of breast cancer cells by altering the expression of cyclin D1 and p21 genes through ER-mediated signaling pathways^20^. These data imply that exposure to phenolic EDCs (BPA, NP and OP) may be related to the prevalence of breast cancer; However, there is currently inadequate information to establish if and under what conditions it directly produces breast cancer. This study employed bioinformatics methods such as network toxicology, machine learning, molecular docking, and molecular dynamics simulation to preliminarily explore the potential toxicity of BPA, NP and OP and their possible pathways of action related to breast cancer, aiming to provide testable hypotheses for subsequent mechanism verification research. We found that traditional toxicology often focuses on the individual action patterns of one or more molecular targets, neglecting the systemic impacts and interconnected toxic pathways^21^. As the toxicity of environmental pollutants typically arises from the disruption of multiple biological molecular networks, this complicates the accurate prediction of the toxic mechanisms of these pollutants in human environments. Therefore, there is a need for more scientific, cost-effective, and efficient new assessment methods to evaluate the potential health risks posed by the increasing number of toxic pollutants in the environment.

Network toxicology is an analytical approach based on network pharmacology and network biology, integrating bioinformatics, big data analysis, and techniques from genomics, proteomics, and metabolomics^21^. This method aids in unraveling complex biological mechanisms without the need for traditional toxicological experiments, characterized by its simplicity, accuracy, and broad applicability. It has been employed in various applications, including environmental health, food safety, drug research, and disease control^22^. Machine learning methods are utilized to predict potential core toxicological targets, while multidimensional bioinformatics analyses emphasize the critical pathogenic roles of these core targets in diseases. Molecular docking is a structure-based computational method that predicts the preferred binding orientation and affinity of small-molecule ligands at the active site of a target protein, and is commonly used in computer-aided drug design, as well as in the study of interactions and affinities between compounds and target genes^23^. In this study, the binding patterns and affinity of phenolic EDCs with key target genes were addressed, and the stability of complexes such as MGLL-BPA was veri-fied through free energy calculations. In recent years, integration methodologies based on bioinformatics and network toxicology have been increasingly used to discover potential molecular links between environmental contaminants and diseases. For example, Zhang et al. found that diphthalate (DEHP) induced nonalcoholic fatty liver disease by interfering with PPARα/γ and fatty acid metabolism pathway using CTD and KEGG enrichment analysis^24^, and Yang et al. integrated network toxicology, single-cell analysis, and molecular docking to analyze polychlorinated biphenyls (PCBs) by altering the PI3K-Akt and MAPK pathways in breast cancer, thereby increasing cancer risk^25^. In addition, Zeng et al. applied network toxicology and molecular docking techniques to reveal that butylated hydroxyisopropanols may promote chronic urticaria flare-ups by regulating natural killer cell-mediated cytotoxic calcium (Ca)^26^. However, comparative systematic bioinformatics research on Phenolic EDCs (BPA, NP, and OP) is still absent, and their shared or particular toxicological networks have not been resolved. Therefore, in this study, we systematically elucidated the potential toxicity mechanisms of BPA, NP, and OP by constructing pollutants-targets-pathways-immunity-functions, combined with molecular docking and kinetic simulation, to provide new theoretical clues for the accurate risk assessment of environmental pollutants and early intervention of related diseases.

## Materials and methods

### Target collection for BPA, NP, and OP

Chemical structures and SMILES notations for BPA, NP, and OP were obtained from the PubChem database (https://pubchem.ncbi.nlm.nih.gov/). Predicted targets for these compounds were identified using different databases, such as the CTD (https://ctdbase.org/), SEA (https://sea.bkslab.org), and Swiss Target Prediction (http://www.swisstargetprediction.ch/about.php), specifying “Homo sapiens” as the species. Target names were standardized using the Uniprot database (https://www.uniprot.org/), and duplicate entries were deleted throughout the merging process to obtain common targets for BPA, NP, and OP.

### Identification of potential breast cancer-related targets

Genes linked to breast cancer were obtained from the GeneCards (https://www.genecards.org) and DisGeNET (https://www.disgenet.org) databases. The retrieved targets were merged, and duplicates were eliminated to identify potential breast cancer-related targets. The intersection of breast cancer-related targets and projected BPA, NP, and OP targets was analyzed to pinpoint potential targets linked to breast cancer risk.

### Functional pathway analysis of potential target

GO and KEGG enrichment analyses provide valuable insights into gene functions, revealing chemical-disease signaling pathways. Enrichment analyses were conducted using the DAVID database (https://david.ncifcrf.gov/), focusing on “Homo sapiens” to find molecular pathways linked to potential genes affected by BPA, NP, and OP in breast cancer. DAVID, a web-based bioinformatics tool, provides comprehensive gene list annotation and analysis features for researchers^27,28^. GO functional analysis encompasses three categories: Biological Process (BP), Cellular Component (CC), and Molecular Function (MF). KEGG pathway enrichment analysis identifies key pathways regulated by chemicals in relation to diseases. The results of GO enrichment and KEGG pathway analyses were organized by P-value and visualized as bubble plots using a bioinformatics server^29^ (https://www.bioinformatics.com.cn/).

### Core target screening using machine learning algorithms

Core targets linked to BPA, NP, and OP-induced breast cancer were identified using two machine learning algorithms: LASSO regression and SVM^30,31^. LASSO is a regression analysis technique that accommodates a large number of covariates, reducing overfitting and enhancing predictive accuracy, making it suitable for high-dimensional data mining and biomarker selection^32^. SVM is a supervised machine learning approach that effectively converts low-dimensional nonlinear data points into linearly separable points in a higher-dimensional space^33^. To enhance model performance, we utilized a nested algorithm, Recursive Feature Elimination (RFE), for optimizing feature selection by identifying the optimal variable combination. The “glmnet” R package (version 4.4.1) was utilized for LASSO, with the following parameter settings: standardization = TRUE, α = 1, family = “gaussian,” nfolds = 3. For SVM-RFE, we used the “mlench” and “caret” R packages, normalizing all data before model training. This nested approach, utilizing multiple algorithms, balances “interpretability” and “predictive power,” facilitating the identification of robust biomarkers with high discriminative capability. Genes identified by both algorithms were classified as core genes.

### Differential expression and diagnostic potential of core genes

We obtained the GEO dataset GSE42568 (https://www.ncbi.nlm.nih.gov/geo/), which comprises 104 breast cancer tissue samples and 17 normal breast tissue samples, providing transcriptomic data for both types of tissues. Data preprocessing and normalization were conducted using R version 4.4.1. Genes exhibiting |log₂FC| ≥ 1 and a corrected P-value (FDR) < 0.05 were identified as significantly differentially expressed. Furthermore, we generated Receiver Operating Characteristic (ROC) curves utilizing the mRNA expression data from breast cancer patients and normal breast tissue samples in the GSE42568 dataset, employing the pROC 1.18.4 package (R version 4.4.1) to assess the diagnostic potential of core targets, and calculated the area under the curve (AUC) to evaluate the diagnostic accuracy of the ROC curves.

### Single gene GSEA enrichment analysis of core genes in breast cancer

GSEA is an effective analytical method frequently employed to evaluate the enrichment of individual genes within a dataset. This method allows for evaluating enrichment by ranking genes based on specific criteria, rather than just focusing on genes with differential expression, and then assessing if genes in the dataset are enriched at the top or bottom of the ranked list. This provides a more thorough evaluation of gene enrichment across the entire dataset and offers deeper insights into gene expression data. To investigate the potential regulatory mechanisms of each core gene in breast cancer more comprehensively, in the study, the “GSEA Enrichment Analysis and Visualization Tool” available on the San Sebastian box platform^34^ was utilized to classify tissue/cell samples (n=) into the Top 25% and Bottom 25% groups based on gene expression levels, serving as the phenotype. Enrichment analysis was executed using the MSigDB collection (C2.CP: KEGG), with a significance threshold established at FDR < 0.25. Additionally, single-gene GSEA enrichment analysis was performed for each core gene.

### Immune infiltration analysis

To assess the alterations in immune cell populations in breast cancer, we employed the GSVA (v1.46.0) R4.4.1 package to conduct ssGSEA^35^. Quantifying infiltration levels of 22 immune cell types in normal and breast cancer cohorts from the GSE 42,568 dataset allowed for a thorough assessment of immune cell infiltration scores in pathological versus normal breast tissues. We performed statistical significance testing using the Wilcoxon rank-sum test. Additionally, we analyzed the relationship between core target genes and immune cell infiltration, generating a correlation heatmap with a significant threshold of *P* < 0.05.

### Molecular Docking

We employed molecular docking methods to investigate the potential binding interactions between core targets and BPA, NP, and OP. The 3-D crystal structures of the target proteins were retrieved from the RCSB Protein Data Bank (PDB) (http://www.rcsb.org/)^36^ and processed in PyMOL 2.5 to remove crystallographic water molecules and co-crystallized ligands. AutoDock Vina 1.5.6 was utilized for molecular docking to examine protein-ligand relationships^37^. Hydrogens were added and partial charges calculated using AutoDockTools-1.5.6 (ADT), after which the macromolecules were stored in PDBQT format. Ligand structures (SDF or MOL2) were retrieved from PubChem or drawn in ChemDraw 21.0, energy-minimized using MMFF94s force field in Chem3D, translated to MOL2, and subsequently prepared in ADT (addition of Gasteiger charges, rotatable bonds assigned, saved as PDBQT). Grid maps were centered on the co-crystallized ligand or on the centroid of the projected active-site residues, with adequate extension to span the entire pocket (spacing = 1.0 Å). Docking simulations were conducted with AutoDock Vina 1.5.6 utilizing the Lamarckian genetic process. For each ligand, 20 separate runs were performed with exhaustiveness = 16; all other parameters were retained at their default settings. The most populated cluster with the lowest binding free energy (ΔG, kcal mol⁻¹) was selected as the representative pose. Complexes were visually evaluated and interaction diagrams created in PyMOL and LigPlot⁺ to verify critical hydrogen bonds, hydrophobic contacts, and π–π stacking.

### Molecular dynamics simulation

Molecular dynamics simulations were conducted with Gromacs 2022.2. Force field parameters were obtained from Gromacs’ pdb2gmx tool and the AutoFF web interface^38^. The receptor protein utilized CHARMM36 force field parameters, while the ligand employed CGenff force field parameters. A 1 nm TIP3P cubic water box was employed for solvation, and ions were introduced for electrical neutrality using the gmx genion tool. The Particle Mesh Ewald (PME) method managed long-range electrostatic interactions with a 1 nm cutoff distance^39^. All bonds were constrained using the SHAKE algorithm, and the integration time step for the molecular dynamics simulation was set to 2 fs via the Verlet leapfrog algorithm. Before the molecular dynamics simulation, the system underwent energy optimization. The energy minimization process consisted of 3000 steps of steepest descent optimization, followed by 2000 steps of conjugate gradient optimization 39. The optimization steps were conducted as follows: initially, the solute was constrained while minimizing the energy of the water molecules; subsequently, counter ions were constrained and energy minimized; finally, the entire system was energy minimized without constraints. The simulation was performed under NPT conditions at a temperature of 310 K (physiological temperature) and constant pressure, with a time step of 2 fs and a simulation duration of 100 ns^40–42^. During the simulation, tools such as g-rmsd, g-rmsf, g-hbond, g-Rg, and g-sasa were employed to calculate the root mean square deviation (RMSD), root mean square fluctuation (RMSF), hydrogen bonds (HBonds), radius of gyration (Rg), and solvent-accessible surface area (SASA), respectively^43^.

## Results

### Potential toxicological targets of BPA, NP, and OP

**Fig. 1:**
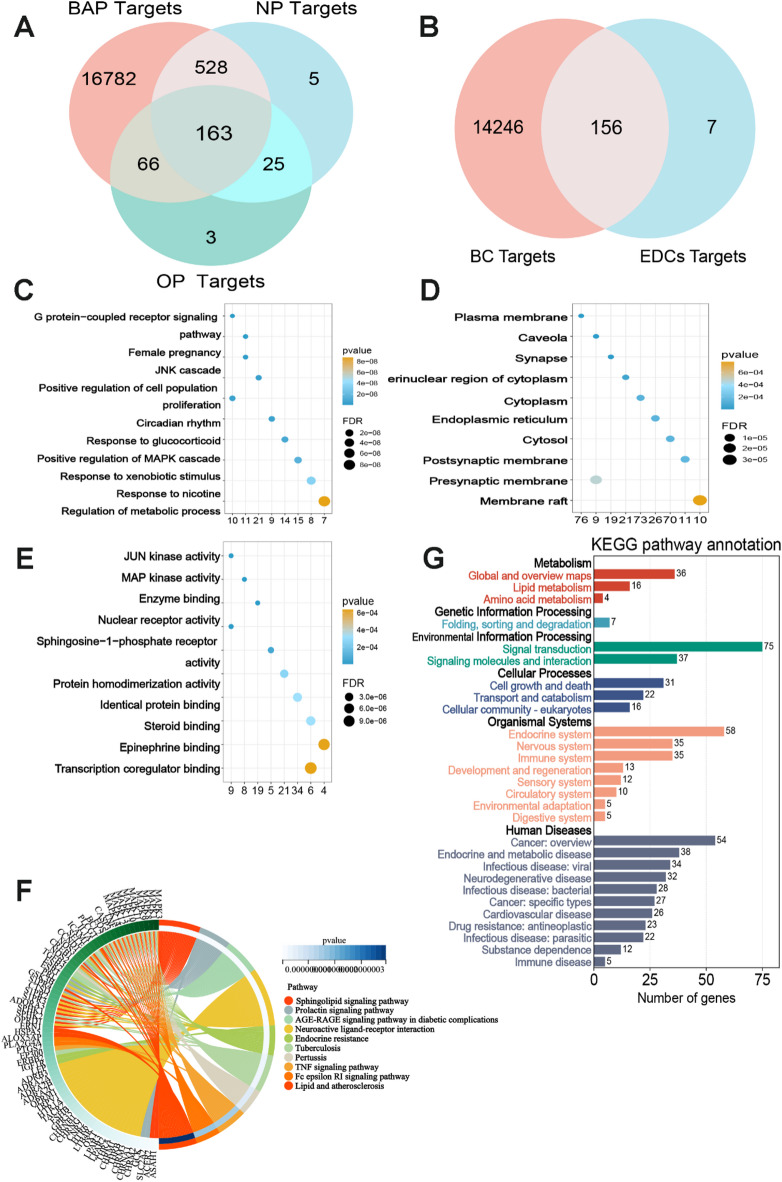
Potential toxicity targets of BPA, NP, and OP, along with their GO and KEGG enrichment analyses.

Network toxicology serves as a sophisticated approach for predicting the mechanisms and targets of environmental pollutants. In this investigation, we employed network toxicology to identify the potential pathogenic targets of BPA, NP, and OP in breast cancer. Data regarding the potential targets of BPA, NP, and OP were sourced from the CTD, SEA, and Swiss Target Prediction databases. Following data integration and deduplication, we identified 163 common targets. As shown in Fig. 1A, BPA, NP, and OP may exert their toxicological effects through these targets. Additionally, after eliminating duplicates, we identified a total of 7202 BC-related target genes from the GeneCards and DisGeNET databases. The overlapping targets of BPA, NP, and OP with genes related to breast cancer are depicted in a Venn diagram in Fig. 1B. In total, we identified 156 common targets, which are regarded as potential targets for breast cancer induced by BPA, NP, and OP.

### Enrichment analysis of potential targets induced by BPA, NP, and OP in breast cancer

We performed enrichment analysis on the 156 overlapping target genes to elucidate the mechanisms underlying BPA, NP, and OP-induced breast cancer. The top 10 GO terms with the lowest false discovery rate (FDR) for each category were visualized. As shown in Fig. 1C-E, in the BP category, the targets were predominantly enriched in the “G protein-coupled receptor signaling pathway,” “Female pregnancy,” and “JNK cascade”. In the CCcategory, “Plasma membrane,” “Caveola,” and “Synapse” were the enriched terms, indicating the potential involvement of these targets in signal transduction. The MF components exhibited significant enrichment in “JUN kinase activity,” “MAP kinase activity,” and “Enzyme binding,” suggesting their potential roles as critical functional modules in cellular signaling and regulation. KEGG pathway enrichment analysis revealed that the identified targets were primarily associated with the “Sphingolipid signaling pathway,” “Prolactin signaling pathway,” and “Endocrine resistance” (Fig. 1F). Further classification of KEGG pathways (Fig. 1G) indicated that these target genes were mainly linked to “Cancer: overview,” “Endocrine system,” “Cell growth and death,” and “Signal transduction.”

A presents the Venn diagram illustrating the overlap among potential toxicity targets of BPA, NP, and OP. B depicts the intersection of potential toxicity gene targets shared by BPA, NP, and OP with genes associated with BC. C-E displays the GO enrichment analyses of the possible toxicity targets induced by BPA, NP, and OP in breast cancer, highlighting the top 10 enriched items categorized by BP, CC, and MF. F illustrates the KEGG pathway enrichment analysis of toxicity targets, focusing on the top 10 enriched pathways. G provides the KEGG secondary classification pathway enrichment analysis. BPA: bisphenol A, NP: nonylphenol, OP: octylphenol, BC: breast cancer.

### Machine learning identification of key targets

**Fig. 2:**
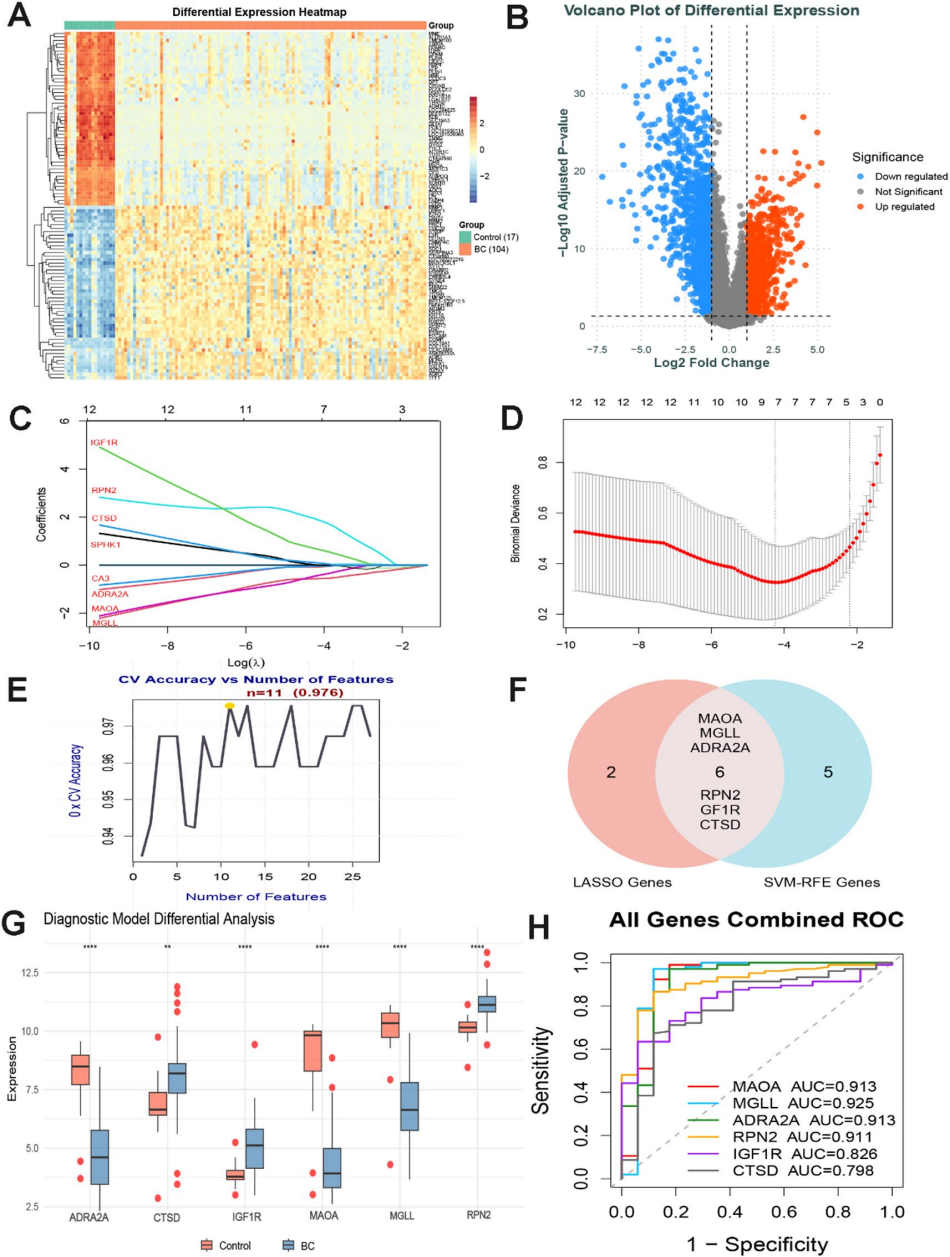
Screening of core targets by machine learning algorithms and construction of gene diagnostic models.

We obtained an RNA-seq dataset (GSE 42568) of breast cancer patients from the GEO database, performed differential gene analysis (Fig. 2A-B), and further applied LASSO and SVM algorithms to identify core target genes from 156 breast cancer-related targets that may be induced by BPA, NP, and OP. Initially, we utilized LASSO logistic regression with a triple cross-validation penalty parameter adjustment process, identifying eight genes as potential core targets (Fig. 2C-D). The SVM regression algorithm further refined this selection to 11 core targets (Fig. 2E). A Venn diagram integrating the results from both methods ultimately identified six distinct core targets associated with BPA, NP, and OP-induced breast cancer: MAOA, MGLL, ADRA2A, RPN2, GF1R, and CTSD (Fig. 2F).

### Expression and diagnostic potential of the six key targets

To validate the expression levels of the core genes, we obtained RNA-seq datasets of breast cancer patients from the GEO database (GSE 42568). The expression of MAOA, MGLL, and ADRA2A was significantly upregulated in breast cancer tissues compared to normal breast tissues, whereas the expression of RPN2, GF1R, and CTSD was significantly downregulated (Fig. 2G). We further constructed ROCcurves and calculated the AUC to evaluate the diagnostic performance of these six core genes. The AUC values for MAOA, MGLL, ADRA2A, RPN2, GF1R, and CTSD were 0.936, 0.925, 0.913, 0.911, 0.826, and 0.798 (Fig. 2H), respectively, indicating that MAOA, MGLL, ADRA2A, and RPN2 possess high translational application potential in the pathogenesis and future diagnosis of breast cancer.

A represents the heatmap of differential gene expression between the breast cancer and normal groups in the GEO dataset GSE42568. B represents the volcano curve of differential gene expression between the breast cancer and normal groups in the dataset GSE42568. C-D represents the LASSO logistic regression technique. The abscissa represents the number of model genes corresponding to different (λ) values, with eight genes identified at the minimum λ value. E denotes the core genes that were identified using the SVM-RFE algorithm, where 11 candidate genes were selected. F denotes a Venn diagram implying the overlap between LASSO and SVM-RFE algorithms, identifying common core targets for BPA, NP, and OP-induced breast cancer. G-H denotes RNA sequence analysis of core gene expression in breast cancer samples versus normal breast tissue. G shows the expression of six key targets in biopsy samples of breast cancer and normal breast tissue from dataset GSE42568. H denotes the diagnostic potential of the six core metrics in distinguishing normal breast from breast cancer samples assessed using ROC curves.

### Single-gene gene set enrichment analysis

**Fig. 3:**
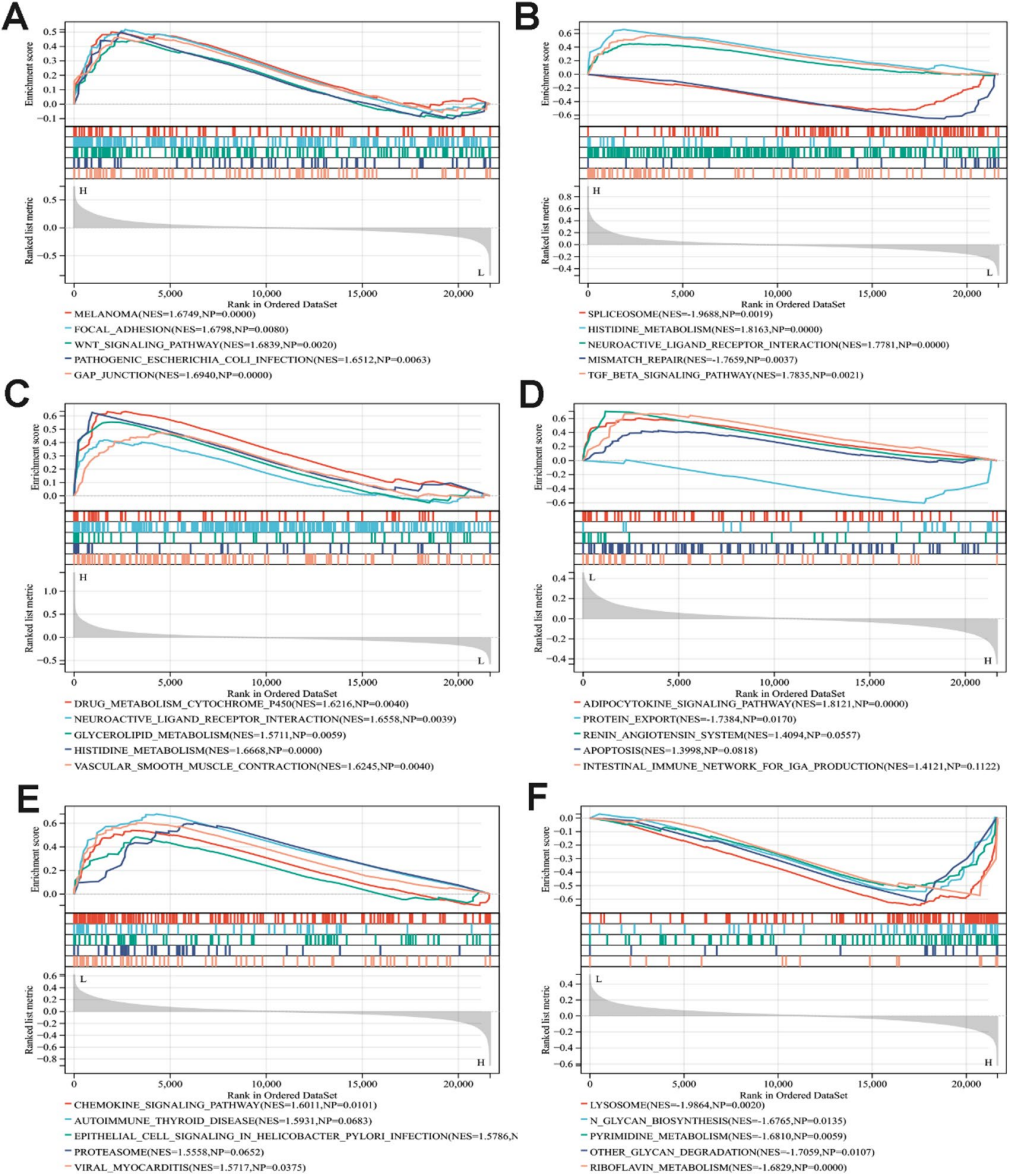
Single-gene GSEA enrichment analysis of core targets.

To investigate the potential regulatory mechanisms of key genes in BC, we performed single-gene GSEA on each of the six core genes using the GSE 42,568 dataset. Our analysis revealed that the expression of these six core genes is significantly linked to various biological pathways (Fig. 3A-F). Notably, pathways such as the Wnt Signaling Pathway (NES = 1.6839, NP = 0.002), TGF-β-Signaling Pathway (NES = 1.7835, NP = 0.0021), Chemokine Signaling Pathway (NES = 1.6011, NP = 0.0101), and Riboflavin Metabolism (NES = 1.6829, NP = 0.0000) are implicated in a range of biological processes, including cell signaling, RNA splicing in tumors, amino acid metabolism, autoimmunity, and the tumor microenvironment. This indicates that BC development is a multifaceted biological process, with the six core genes potentially influencing BC progression through the regulation of distinct pathways. Importantly, several immune-related signaling pathways were found to be significantly enriched, leading us to reasonably speculate that the expression of core genes may be closely associated with the immune response in BC.

A-F represent the GSEA enrichment analysis plots for the core genes MAOA, MGLL, ADRA2A, RPN2, GF1R, and CTSD, respectively. The x-axis indicates the rank of genes in the ordered dataset (Rank in Ordered Dataset), while the y-axis represents the enrichment score (ES). The upper curve illustrates the enrichment trend of the core genes in specific pathways, where a positive peak value suggests significant enrichment and potential activation of these pathways by the gene, whereas a negative value indicates the opposite. The middle section displays the ranking and position of the core genes. The lower shaded area represents the indicator rank score. The bottom lists the Top-five pathways, with colors corresponding to the upper curves.

### Immune infiltration of core genes and correlation with immune cells

**Fig. 4:**
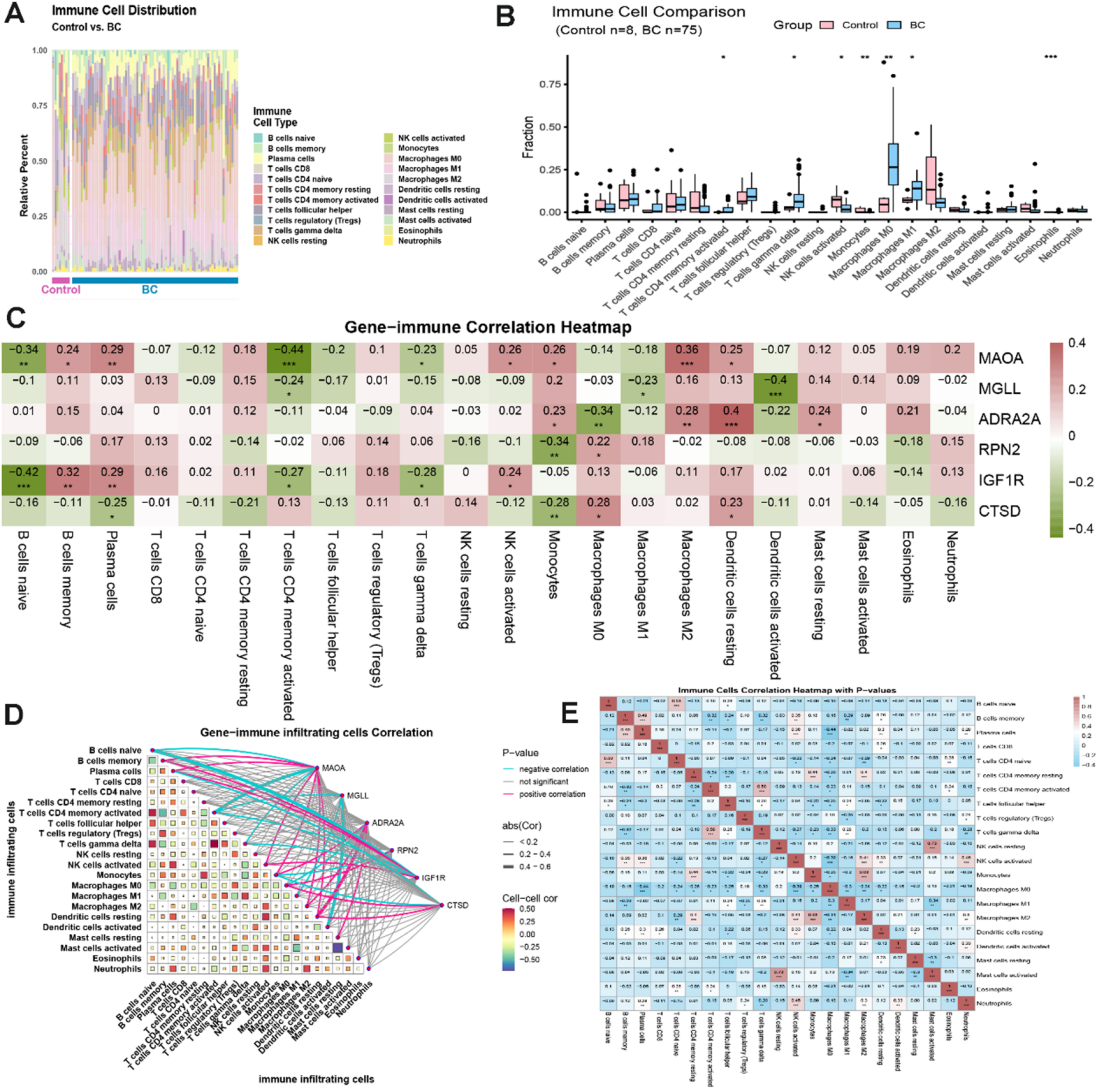
Immune infiltration analysis of breast cancer and normal breast tissues in dataset GSE42568.

To further elucidate the extent of immune cell infiltration in BC, we utilized ssGSEA to assess the composition of 22 immune cell types in normal breast tissue and breast cancer samples from the GSE 42,568 dataset, along with their correlation to the six core genes (Fig. 4A-B). Our findings indicated statistically significant differences in the expression of nine immune cell types between the normal and BC groups, specifically activated CD4 memory T cells, gamma δ T cells, activated NK cells, monocytes, M0 macrophages, M1 macrophages, M2 macrophages, activated mast cells, and eosinophils. This suggests that immune cell infiltration levels are crucial in the onset and progression of BC. Additionally, we conducted correlation analyses to investigate the relationship between core genes and immune cell infiltration in BC. We subsequently examined the association between the six core targets and immune cell infiltration scores (Fig. 4C-D). The expression levels of MAOA, MGLL, ADRA2A, RPN2, GF1R, and CTSD were positively correlated with the infiltration scores of memory B cells, plasma cells, M2 macrophages, and resting dendritic cells. Conversely, these core genes displayed negative correlations with the infiltration scores of naive B cells, activated CD4 memory T cells, and activated dendritic cells. Notably, MAOA, MGLL, and ADRA2A exhibited positive correlations with monocyte infiltration scores while showing negative correlations with M0 macrophages. In contrast, RPN2, GF1R, and CTSD demonstrated the opposite relationship. These findings suggest that the expression of key genes is intricately linked to extensive immune cell infiltration in BC, indicating that these genes may influence the occurrence and progression of BC by modulating the degree of immune cell infiltration. We also identified specific correlations among different immune cell types (Fig. 4E). For instance, resting NK cells were positively correlated with activated mast cells, while exhibiting negative correlations with most other immune cells. Activated CD4 memory T cells were positively correlated with activated gamma δ T cells but negatively correlated with resting and activated NK cells, as well as monocytes (M0, M1, M2) and dendritic cells (resting, activated).

A-B represent ssGSEA of 22 different immune cell types in breast cancer tissues and normal breast tissues. C-D indicates the correlation between core target genes and immune cell infiltration in breast cancer. E demonstrates the correlation study among immune cell types in breast cancer tissues.

### Molecular Docking analysis of compounds and core target molecules

**Fig. 5:**
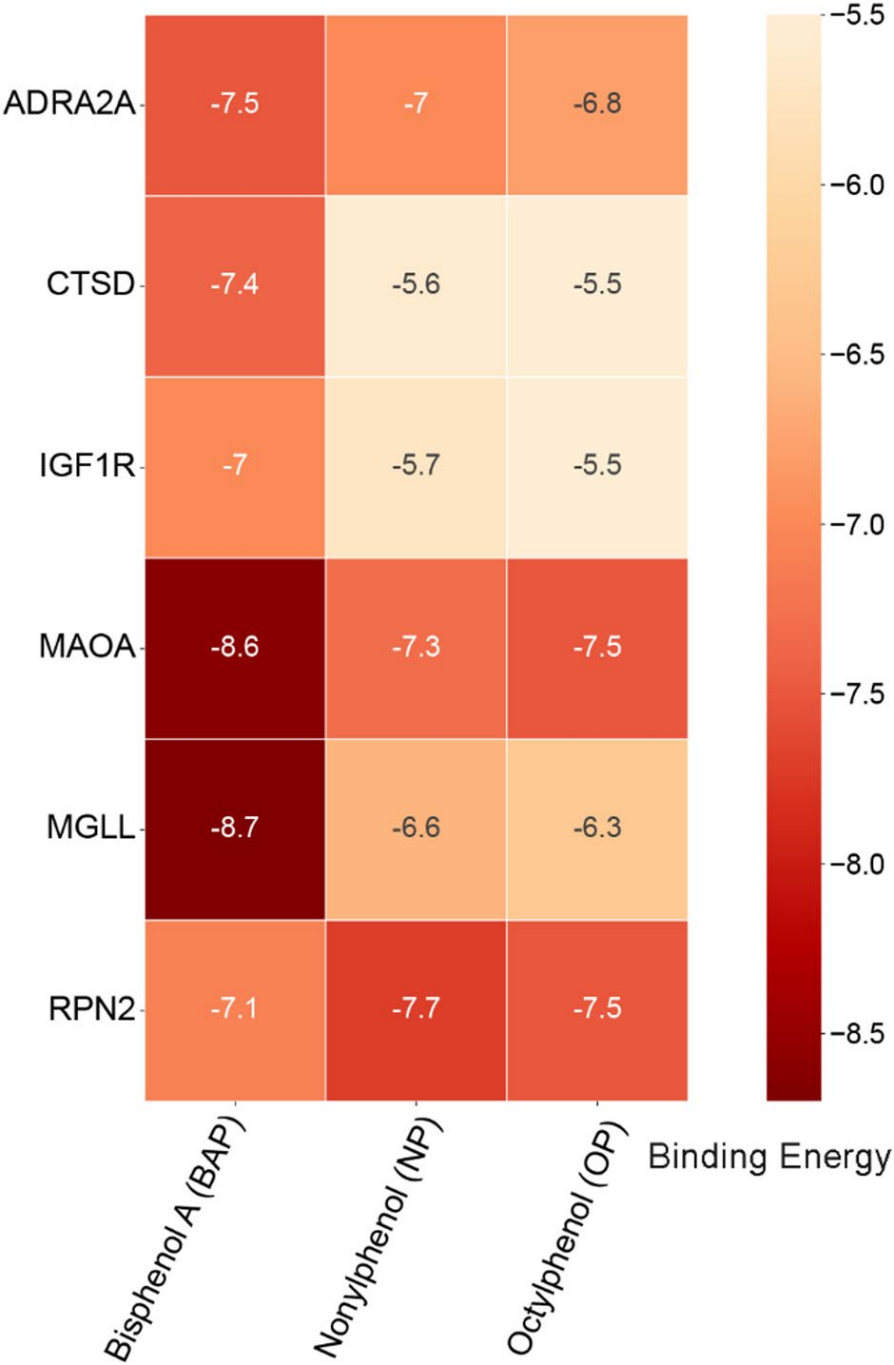
Molecular docking heatmap.

**Fig. 6:**
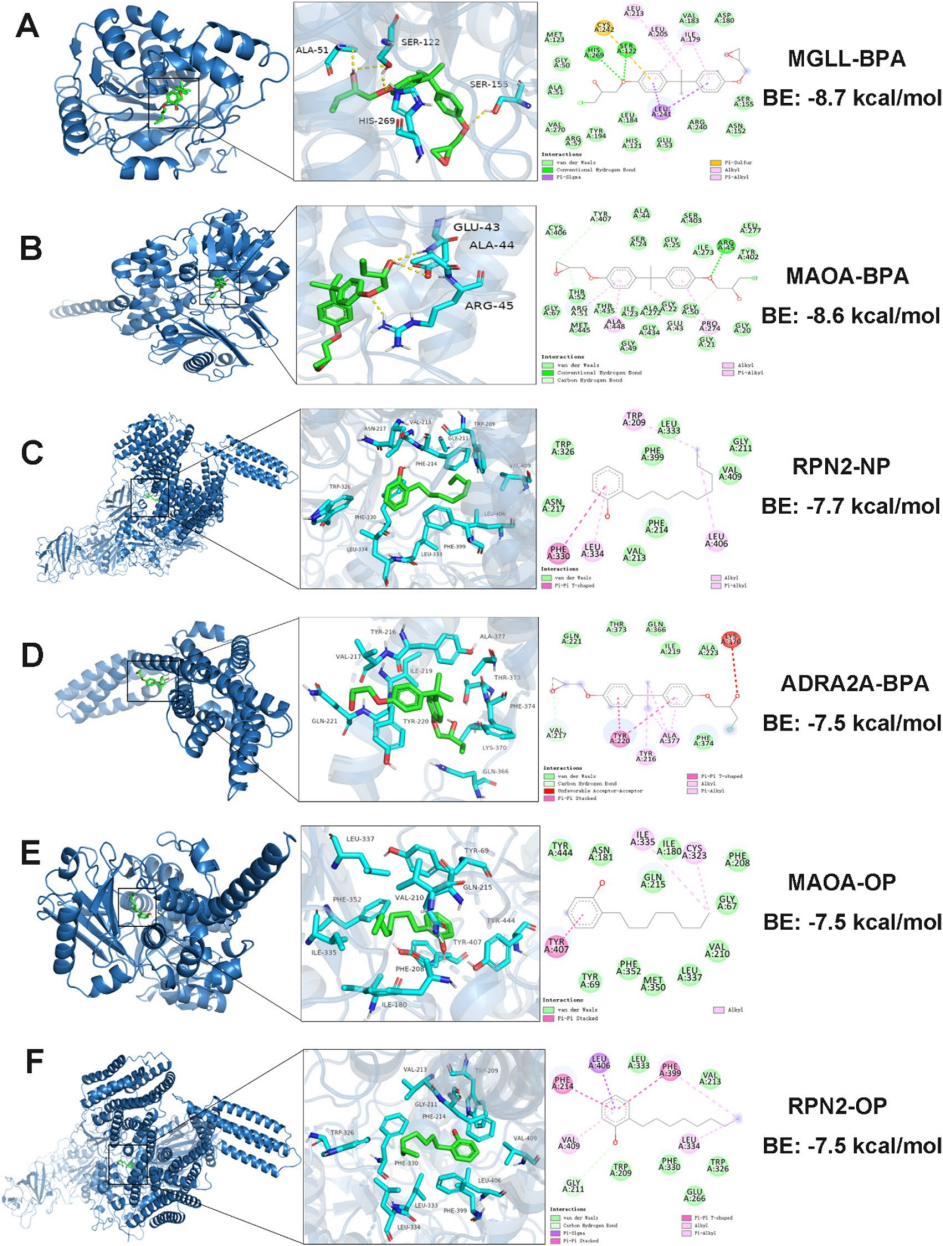
Molecular docking results demonstrate the binding affinity of the compound to each core target protein.

We conducted a molecular docking analysis to assess the interactions between BPA, NP, and OP with six core target proteins: MAOA, MGLL, ADRA2A, RPN2, GF1R, and CTSD. A binding energy of less than 0 kcal/mol indicates that the receptor and ligand can bind spontaneously without the need for external energy. Generally, a binding energy below − 5.0 kcal/mol suggests favorable binding activity, while values below − 7.0 kcal/mol indicate strong binding activity; lower binding energy correlates with stronger binding activity and higher affinity, leading to more stable conformations^44^. The binding energies obtained from the molecular docking analysis are presented in the heatmap in Fig. 5. The binding energies of BPA, NP, and OP with the six core target proteins are all below − 5 kcal/mol, suggesting potential binding capabilities. Notably, the binding energies of BPA with all six core target proteins are below − 7 kcal/mol, indicating a high binding affinity. As shown in Fig. 6A, the strongest affinity was observed between BPA and MGLL, with a binding energy of −8.7 kcal/mol. The SER122 and HIS269 residues on the MGLL receptor form hydrogen bonds with BPA. Additionally, the LEU213, LEU205, and ILE179 residues on the MGLL receptor establish hydrophobic interactions with BPA. Furthermore, the ASP180, VAL183, MET123, GLY50, ALA51, VAL270, ARG57, LEU184, HIS121, GLU53, ARG240, ASN152, and SER155 residues on the MGLL receptor contribute to van der Waals interactions with BPA, while the CYS242 residue engages in Pi-Sulfur interactions, and the LEU241 residue participates in Pi-Sigma interactions with BPA. Figure 6A-F is a visual display of the top 6 docking energy rankings. (Supporting Information Text S1 presents the detailed docking sites with the top 6 docking capabilities.)

The molecular docking heatmap illustrates the molecular docking energies of compounds BPA, NP, and OP with each core target protein. Lower values indicate more stable binding.

A displays the 3D and 2D visualizable molecular docking outcomes of MGLL and BPA. B exhibits the 3D and 2D visualizable molecular docking results of MAOA and BPA. C demonstrates the 3D and 2D visualizable molecular docking findings of RPN2 and NP. D showcases the 3D and 2D visualizable molecular docking results of ADRA2A and BPA. E illustrates the 3D and 2D visualizable molecular docking outcomes of MAOA and OP. F presents the 3D and 2D visualizable molecular docking results of RPN2 and OP. BAP: Bisphenol A; NP: Nonylphenol; OP: Octylphenol.

### Molecular dynamics simulation and binding free energy assessment

**Fig. 7:**
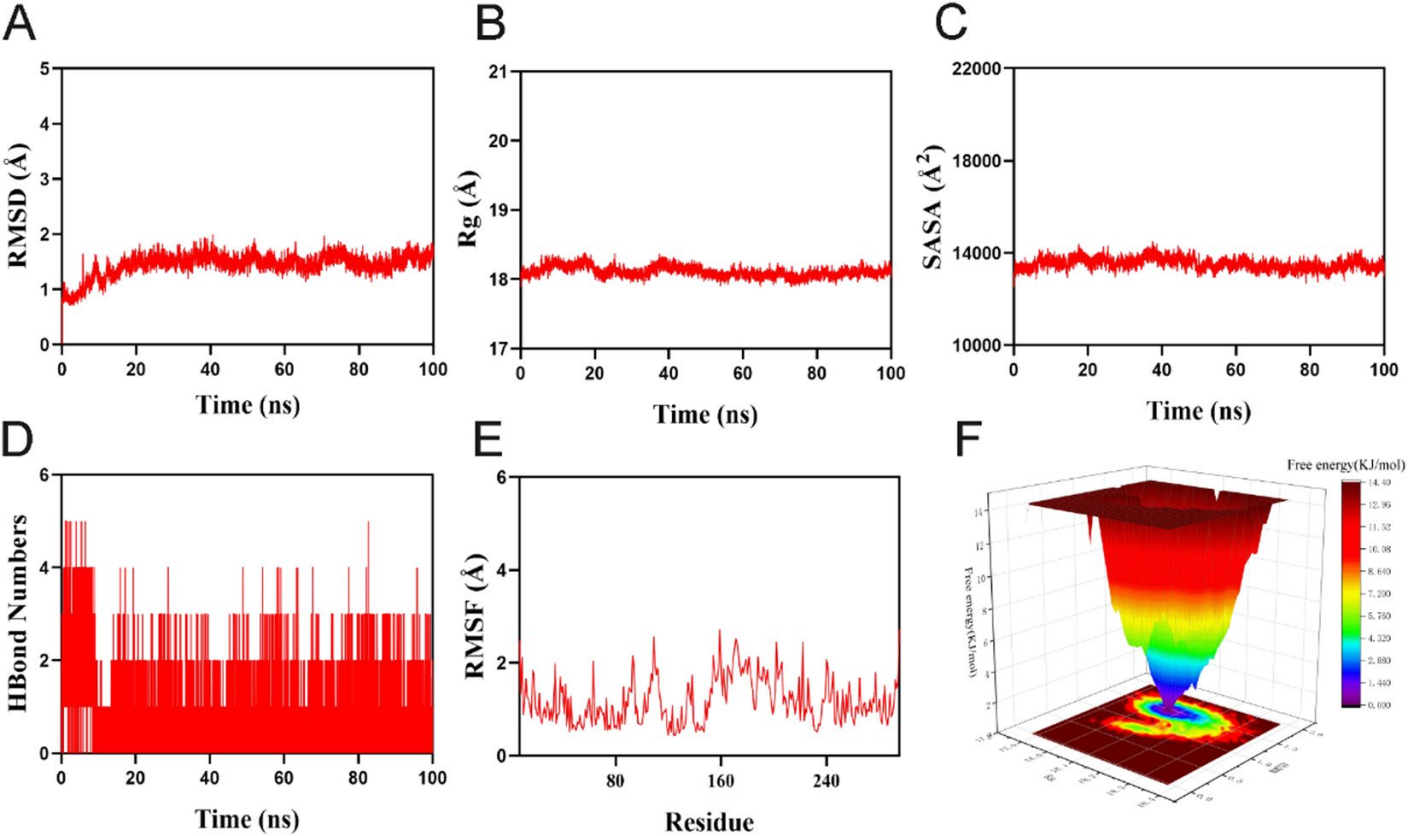
Molecular dynamics simulation of the MGLL-BPA complex.

While the molecular docking analysis employed a semi-flexible docking approach, it did not account for the flexibility of protein structures, temperature, pressure, and solvent effects. To further investigate the stability of the protein-ligand interactions, we performed molecular dynamics (MD) simulations of the MGLL-BPA complex. The RMSD serves as a reliable indicator of the conformational stability of both the protein and ligand, as well as the extent of deviation of atomic positions from their initial states. Smaller deviations signify better conformational stability. Thus, we evaluated the equilibrium of the simulation system using RMSD. As shown in Fig. 7A, the complex system reached equilibrium after 20 ns, fluctuating around 1.6 Å, indicating that the small molecule maintains high stability when bound to the target protein.

The Rg describes changes in overall structure and characterizes the compactness of the protein structure; larger Rg changes indicate greater system expansion. The complex exhibited relatively stable fluctuations during motion, suggesting that the small molecule-target protein complex did not experience significant expansion or contraction during the simulation (Fig. 7B).

The SASA serves as an indicator for assessing the surface area of proteins. This simulation calculated the SASA between the target protein and the small molecule (Fig. 7C), revealing no significant change in the SASA of the complex after receptor-ligand binding, indicating that the ligand’s binding minimally impacts the protein structure. Hydrogen bonds are crucial for ligand binding to proteins.

The number of hydrogen bonds between small molecules and target proteins in the kinetic process is shown in Fig. 7D. The number of hydrogen bonds varied from 0 to 5, with the complex typically exhibiting around 3 hydrogen bonds, indicating a favorable hydrogen bond interaction between the ligand and the target protein.

The RMSF reflects the flexibility of amino acid residues in the protein. As shown in Fig. 7E, the RMSF values for this complex are relatively low (mostly below 2.3 Å), indicating low flexibility and high stability. The free energy topography is a good representation of the protein free energy changes during simulation (Fig. 7F).

In conclusion, the complex system demonstrates stable binding, with effective hydrogen bond interactions, suggesting that the small molecule successfully binds to the target protein.

A represents the RMSD values of the MGLL-BPA complex over time. B represents the Rg values of the MGLL-BPA complex over time. C represents the SASA values of the MGLL-BPA complex over time. D represents the HBonds values of the MGLL-BPA complex over time. E represents the RMSF values of the MGLL-BPA complex. F represents the free energy distribution, where the red, green, blue, yellow, and orange molecular structures in the figure correspond to the ligand small molecule structures at five time points: 0, 25, 50, 75, and 100 ns, respectively.

## Discussion

EDCs are substances from external sources that interfere with the endocrine system’s function, causing harmful effects on organisms, which were categorized by application, chemical composition, and structural properties^45–47^. They fall into various groups, including persistent organic pollutants (POPs), plasticizers, additives, polycyclic aromatic hydrocarbons (PAHs), pesticides, and pharmaceuticals and personal care products (PPCPs)^46^. They are commonly found in pesticides, products for children, materials for food packaging, electronics, construction, personal care items, medical tubing, antimicrobial agents, textiles, and apparel^48^. EDCs are widespread in the environment, detected in various water sources such as surface water, groundwater, drinking water, wastewater, and sediments, causing pollution and posing a significant risk to human health^34^. Scientific attention towards environmental EDCs has increased in the last fifty years. Similar to hormones, EDCs exhibit intricate dose-response relationships, can function at very low levels, and demonstrate synergistic impacts^34^. Because of their enduring and accumulative characteristics in humans and the environment, these substances have long-term effects in humans when exposed for a prolonged period of time and have highly toxic and carcinogenic properties. In both in vivo and epidemiological studies, early living or chronic exposure to multiple EDCs is directly associated with an increased incidence of breast tumors. Prenatal administration of diethylbestrol (DES) or BPA to rodents induces ductal hyperplasia and adenocarcinoma-like lesions in the breast. When the animals are challenged by the chemical carcinogen 7,12-Dimethylbenz[a]anthracene (DMBA), the tumor incidence further increases, indicating that EDCs play a “first blow” role in the occurrence of breast cancer^49,50^. Consistently, women exposed to DES in utero have a 30–40% increased risk of breast cancer after the age of 40^51^. Mechanically, EDCs promote the proliferation of mammary epithelial cells through receptor-dependent and receptor-independent pathways. The ER-dependent pathway: Low sodium concentrations of BPA, NP bind to ERα and membrane receptor ER/GPR30, rapidly activate cyclin D1 and PI3K-AKT signaling pathways, and accelerate the G1/S transition of human MCF-7 breast cancer cells^52^. In addition, BPA exposure during pregnancy can continuously reduce the H3K27me3 levels in the ER-binding regions within the HOTAIR and Wnt4 promoters in the mammary glands of mice and increase the H3K4me3 levels, leading to persistent overexpression of proliferation-promoting genes, which can even be detected in the F3 generation^53^. Overall, these animal and mechanism data provide a direct causal relationship between EDCs exposure and breast cancer development, rather than merely a simple indirect interaction with ER^54^.

Prior research has focused primarily on EDCs rather than phenolic EDCs. The toxicity targets and mechanisms of action of BPA, NP, and OP, the most commonly found phenolic EDCs in environmental and human biomonitoring studies, have not been adequately documented^55^. In our study,17,539 BPA target genes, 721 NP target genes, and 257 OP target genes were acquired from CTD, SEA, and Swiss Target Prediction databases. The overlap of these genes with breast cancer genes identified 156 potential toxicological targets linked to BPA, NP, and OP-induced breast cancer. Enrichment analyses revealed their strong association with various biological functions, including reproductive endocrinology, inflammation, immune cell cycle, apoptosis, and cancer-related processes. Prior research has suggested that BPA induces distinct physiological responses by interacting with nuclear and membrane steroid receptors such as ER, PR, AR, and PPAR^56^. BPA has been proven to enhance the growth of breast cancer cells by facilitating communication between amphiregulin and receptor-mediated pathways in breast cancer cell lines expressing ER^57^. However, sufficient data suggest that the effects of BPA on breast cancer can be independent of interactions with ER in cancer cells, e.g., through immunomodulation^58^. Due to its phenolic structure, NP has been shown to act as a G protein-coupled ER (GPER) agonist, thus interfering with estrogen signaling pathways, and it is crucial to elucidate the possible role of NP in the etiology of estrogen-dependent tumors^59,60^ Additionally, NP can stimulate the growth of ER-positive epithelial cells, potentially supporting tumorigenesis in estrogen-sensitive cancers^61^. Another investigation found a direct association between NP exposure and DNA harm in a laboratory setting. Given the significance of DNA damage in cancer development^62^. OP is categorized as an alkylphenol synthetic chemical. OP binds to the ER, inducing abnormal activation of ER signaling, leading to increased expression of oncogenes. Prolonged exposure is linked to the onset of ER-positive breast cancer^63^. It has been hypothesized that OP may cause cancer-proliferative effects on estrogen-dependent cancers (e.g., breast and ovarian cancers) like that of E2^64^.

To further identify the role of target genes in BC. We used the DAVID database for enrichment analysis, and the results advised that it was closely correlated to biological functions such as reproductive endocrinology, inflammation, immune cell cycle, apoptosis, and cancer-related. We obtained the BC-related dataset GSE42568 from the GEO database and used 2 different machine learning algorithms. The LASSO algorithm and SVM-RFE model were employed to identify six well-defined core targets potentially associated with the induction of breast cancer by BPA, NP, and OP. MAOA, MGLL, ADRA2A, RPN2, GF1R, and CTSD, we examined their expression levels in breast cancer tissues to further investigate the relevance of these six core targets in breast cancer. Our results suggested that the expression of MAOA, MGLL, and ADRA2A was significantly up-regulated in breast cancer tissues compared to normal breast tissues, and conversely, the expression of RPN2, GF1R, and CTSD was significantly down-regulated. In addition, we constructed ROC curves to assess the potential diagnostic value of these six core indicators for breast cancer. The ROC curve analysis suggested that the AUC of four of these core indicators, MAOA, MGLL, ADRA2A, and RPN2, was greater than 0.9%. These results indicate that the four primary indicators exhibit robust discriminatory capability and are suitable biomarkers for diagnosing breast cancer. This underscores the crucial involvement of the identified core targets in the development of breast cancer.

In addition, single-gene GSEA enrichment analysis suggested that these six core genes could affect breast cancer development through multiple biological pathways, including cell signaling, RNA splicing in tumors, amino acid metabolism, autoimmunity, and tumor microenvironment. Among them, immune-related signaling pathways were also found to be enriched, suggesting that immune regulation plays a key role in breast cancer pathogenesis. Complex quantitative and qualitative differences in the immune populations of the immune tumor microenvironment (TME) in different subtypes of breast cancer lead to a dual role of different elements of the immune TME in limiting and promoting tumor growth^65^. Favorable prognosis is linked to immune-promoting factors such as functional CD4 T cells, CD8 T cells, NK cells, B cells in tertiary lymphoid structures, and plasma and dendritic cells^66^. Conversely, poor prognosis is associated with immunosuppressive factors like regulatory T cells, neutrophils, and MHC-II expression-reduced macrophages expressing triggering receptors on myeloid 2 (TREM2)^66^.

In this study, ssGSEA was utilized to examine the existence of 22 different types of immune cells in RNA-SEQ data derived from normal breast tissues and breast carcinoma tissues. In addition, the link between six main gene targets, namely MAOA, MGLL, ADRA2A, RPN2, GF1R, and CTSD, and the invasion of immune cells in breast tumor tissues was also investigated. The results revealed that the expression levels of these core genes were highly linked with the infiltration abundance of distinct immune cells in the TME. Including activated CD4 memory T cells, γδ T cells, activated natural killer cells (NK cells), M0 macrophages, M1 macrophages, and M2 macrophages. In recent years, multi-omics studies have continuously revealed that the progression of breast cancer not only depends on the genetic and epigenetic changes of cancer cells themselves, but is also closely related to the metabolic-immune interaction network in the tumor immune microenvironment (TIME)^67–70^. The six core genes together influence the infiltration pattern and functional polarization of CD4⁺ memory T cells, γδ T cells, activated NK cells, and M0/M1/M2 macrophages by controlling metabolic axes such as neurotransmitters, lipids, glycoproteins, and proteolysis. This will have a synergistic effect on immune escape, metastasis, and dissemination, and treatment tolerance of breast cancer^71–73^.

Most CD8 and CD4 T cells in breast cancer are effector memory cells + +^74^. B cell clusters in tertiary lymphoid structures are mainly enclosed by CD4 T cells and CD8 T cells. Tumor-infiltrating B cells can present antigens to CD4 T cells to enhance anti-tumor immunity^75^. This indicates that the significance of immune reactions in the progression of breast cancer should not be overlooked. Although BC exhibits immunosuppressive characteristics, TNBC displays immunogenic properties and is abundant in tumor-infiltrating lymphocytes that activate the immune response^76,77^. Several studies have established that immune infiltration has a strong impact on breast cancer progression, and in particular, T-cell infiltration affects patient survival in all breast cancer subtypes^78,79^ Atezolizumab and pembrolizumab, immune checkpoint inhibitors targeting the PD-1/PD-L1 receptor on T cells, are key in TNBC treatment and exhibit encouraging clinical effectiveness^80^. Preliminary evidence supports the involvement of tumor-associated macrophages (TAM) in the progression and metastasis of breast cancer^81^. Macrophages are traditionally categorized into two types: M1, with anti-cancer effects, and M2, which support tumor development and spread^82,83^. A study suggests that TNBC can promote the transformation of M1 macrophages to M2 macrophages through the secretion of increased amounts of granulocyte colony-stimulating factor (G-CSF)^84^. These findings suggest a change in the immune microenvironment of breast tumors and provide a potential avenue for research. We also analyzed the correlation of six core indicators with immune cell infiltration. We reported that the expression levels of MAOA, MGLL, ADRA2A, RPN2, GF1R, and CTSD were positively correlated with B cell memory, Plasma cells, Macrophages M2, and Dendritic cells resting infiltration scores. In contrast, negative correlations were shown between these core genes and naïve B cells, memory activated CD4 T cells, and Dendritic cells, and activated infiltration scores. BPA, NP, and OP might impact the development of breast tumors by altering the immune environment, potentially enhancing the spread of breast cancer through increased infiltration of M2 macrophages.

The molecular docking analyses revealed that BPA, NP, and OP exhibited binding energies below − 5 kcal/mol towards the six core target proteins, suggesting their potential binding capability. Notably, BPA demonstrated binding energies below − 7 kcal/mol with all six core target proteins, highlighting its strong binding affinity. Particularly, BPA displayed the highest binding affinity with MGLL (−8.7 kcal/mol), as supported by subsequent molecular dynamics simulations. Static molecular docking can only provide a “snapshot” binding conformation and cannot reflect the thermal variations at physiological temperatures. Our 100 ns RMSD trajectory demonstrates that the MGLL-BPA complex converges to the 1.6 A platform after 20 ns, as reported by Hazard et al. “Endocrine disruptor -PPARγ complex RMSD < 2.0 Å is regarded as high stability and the threshold is consistent^85^. This study supports the prediction of network toxicology that “MGLL is a high-affinity target for BPA”. Additionally, we observed that the Rg fluctuated throughout the process at 18 A, and the SASA changed < 1.5%, with three continuous hydrogen bonds in the trajectory, and the RMSF of the MGLL cap region is mostly below 2.3 A, indicating that BPA binding does not trigger a wide range of conformtional changes in MGLL, but rather reduces its flexibility through “kinetic clamping” of the cap region, thereby inhibiting substrate entry^86^. This work employs MGLL-BPA as an example to demonstrate that only when the five indications of RMSD, Rg, SASA, Hydrogen Bond, and RMSF simultaneously meet the threshold can the target be moved into the subsequent experimental verification step. This approach can be extended to other phenolic EDCs to boost screening efficiency and reduce experimental expenses. In conclusion, molecular dynamics simulations not only suggested the high stability and specificity of the MGLL-BPA complex under physiological conditions but also clarified at the atomic level the microscopic mechanism by which BPA restricts the dynamics of the MGLL cap region through a hydrogen bond network and thereby interferes with lipid metabolism.

The MGLL gene encodes a key lipolytic enzyme belonging to the serine hydrolase superfamily, whose main function is to break down monoacylglycerol into free fatty acids and glycerol, which is involved in energy metabolism and lipid signaling regulation^87^. Research has demonstrated that MGLL exhibits high expression in primary tumors and invasive cancer cells, where it controls fatty acid networks abundant in oncogenic signaling lipids. This regulation facilitates tumor growth, migration, invasion, and survival in vivo.In addition, high MGLL expression is associated with M2-type macrophage polarization, a polarized phenotype that suppresses anti-tumor immune responses and promotes tumor immune escape^88^. In breast cancer, high MGLL expression is associated with tumor aggressiveness and poor prognosis. It supports rapid tumor proliferation and metastasis by promoting fatty acid metabolism, which provides energy and synthetic raw materials for cancer cells^89^. Therefore, it is reasonable to speculate in our study that putative BPA may promote immune escape from breast malignancy by promoting high MGLL expression, which in turn promotes macrophage differentiation toward the M2 type.

The MAOA gene, responsible for neurotransmitter metabolism, also influences the immune system^90^. The expression of MAOA in breast cancer tissues correlates with the tumor’s immune microenvironment^91^. High MAOA expression was associated with TME, which may promote immune escape and tumor progression by regulating TAM polarization^92^. Specifically, it may be related to the high expression of MAOA in tumor-infiltrating CD8 + T cells, which correlates with T-cell depletion from the suppression of the anti-tumor immune response^93^. Moreover, elevated MAOA levels in melanoma hinder the effectiveness of PD-1 blockers, indicating that MAOA could serve as a potential target to overcome resistance to immunotherapy^94^. Clinical data analysis revealed an inverse relationship between increased MAOA gene expression in breast cancer individuals and decreased overall survival rates^95,96^. The results of bioinformatics analysis in this study also suggested that the high expression status of the MAOA gene is associated with breast cancer tissues.

The ADRA2A gene encodes an α2A-adrenergic receptor, a G-protein-coupled receptor primarily regulating the sympathetic nervous system. Recently, its involvement in breast cancer advancement, modulation of the immune microenvironment, and response to immunotherapy has been recognized^91^. In a combined microarray analysis covering 1,988 primary breast cancer cases, high ADRA2A expression was significantly associated with metastasis-free recurrence^97^. In a different case-control study from 2015, the ADRA2A gene polymorphism was linked to the severity, rather than the likelihood, of breast cancer^98^. Therefore, further studies are needed to investigate the relationship with ADRA2A in BC risk or prognosis. Recently, some researchers found that ADRA2A exhibited potent antitumor activity in multiple immunoreactive tumor models, and ADRA2A agonists can induce potent antitumor immune responses by directly acting on macrophages and enhancing the activation effect on CD4+/CD8 + T cells, which significantly improves the clinical efficacy of tumor immunotherapy, suggesting that ADRA2A is a key activation target for immunity^99^.

RPN2, an integral membrane protein in the rough endoplasmic reticulum, is a crucial part of the N-oligosaccharyltransferase complex (OST), essential for N-glycosylation of new polypeptides^100^. Recent research has revealed that RPN2 is crucial in breast cancer’s onset, progression, drug resistance, and spread. Additionally, it might impact tumor immune evasion through immune molecule glycosylation control^101^. RPN2 is a potential regulator of breast cancer stem cell characteristics. High expression of RPN2 is associated with breast cancer stem cell (BCSC) characteristics, which may be mediated by Wnt/β- catenin or GSK3β signaling pathway to maintain stem cell-like phenotype and enhance tumor initiation and metastatic potential^102^. Moreover, RPN2 regulates the localization of CD63 on the cell membrane through mediated glycosylation of CD63, thereby enhancing the invasiveness and malignancy of breast cancer cells^103^. RPN2 boosts resistance to docetaxel in breast cancer cells by controlling glycosylation and membrane positioning of P-glycoprotein (P-gp) 85, potentially contributing to the emergence of BCSC cells^104^. Considering that BCSCs are usually of low immunogenicity and resistant to immune attack, this mechanism may also be closely related to immune escape. Due to the multiple pro-oncogenic effects of RPN2 in breast cancer, researchers have proposed that it may serve as a new target for interfering RNA therapy. By targeting RPN2, it may not only be possible to reverse chemotherapy resistance^104^ but also enhance the immune system’s ability to recognize and clear tumors, providing a new strategy for combined immunotherapy. Therefore, RPN2 is not only a potential biomarker for breast cancer prognosis, but also expected to be a new target for combining chemotherapy and immunotherapy.

GF1R is a transmembrane tyrosine kinase receptor that plays multiple roles in breast cancer development, progression, and regulation of the immune microenvironment. GF1R, commonly found in a phosphorylated state as pGF1R, is prevalent across various subtypes of breast cancer, with significant variation in overall expression levels observed. Expression levels differ markedly among breast cancer subtypes, with elevated levels noted in Luminal A breast cancer, typically linked to a more favorable prognosis. In a clinical trial, GF1R activation status (pGF1R/IR) was associated with a favorable response to neoadjuvant treatment regimens in patients with hormone receptor-positive breast cancer^91,105^. In contrast, in triple-negative breast cancer, IGF1R expression is lower and associated with poorer survival, and its reduced expression is associated with the formation of an immunosuppressive microenvironment^106^, including elevated expression of IL-6 and CCL2, and increased infiltration of CD11b+ monocytes, which promotes tumor metastasis and immune escape^107^. In general, elevated GF1R signaling pathway activity is closely associated with increased tumor proliferation, anti-apoptosis, and invasiveness. This subtype dependence suggests that GF1R may serve as a prognostic marker and therapeutic target.

CTSD, an aspartic acid protease found in lysosomes, plays various roles in breast cancer progression, including metastasis, immune regulation, and targeted immunotherapy^108^. It is overexpressed in breast cancer cells and released into the tumor microenvironment as an inactive form, contributing to tumor growth, fibroblast proliferation, angiogenesis, and metastasis^109^. CTSD deficiency delays breast carcinogenesis and induces tumor cell quiescence by a mechanism that inhibits the mTORC1 signaling pathway and induces cells into a reversible quiescent state^110^. CTSD was incorporated into macrophage-associated marker gene models for predicting prognosis in TNBC patients. Elevated CTSD expression in high-risk patients, accompanied by elevated T-cell inflammation scores and up-regulation of immune checkpoint molecules (e.g., PD-1, LAG3, and IDO1), suggests that CTSD may be involved in immune escape and is expected to serve as an immunotherapy target^111^. Its expression level is closely related to prognosis and immunotherapy response in breast cancer patients.

Although the machine learning process identified six robust predictors, the remaining 150 differentially expressed genes may also regulate the pathways of breast cancer occurrence. A comprehensive functional analysis of these additional candidate genes is necessary, but it is beyond the scope of this study. All six identified key genes are crucial in breast tumor development. While the study did not directly show how these genes induce breast cancer, further research is necessary to ensure their role. This study enhances our understanding of phenolic EDC hazards and guides future research. With the widespread use of phenolic EDCs due to rapid industrial growth, evaluating their presence in everyday products for safe exposure levels is essential. Strengthening regulatory standards can minimize EDC exposure, particularly in products in direct contact with the body, like beverage bottles, food containers, and cosmetic packaging. Regulatory bodies should address the introduction of EDCs from common consumer products into the body, especially in vulnerable groups like infants, pregnant women, and children. Promoting safer alternatives may be necessary to reduce exposure levels.

By using network toxicology, bioinformatics, molecular alignment, and molecular dynamics simulation techniques, this study not only explored the potential toxic targets and mechanisms of action of phenolic EDCs, including BPA, NP, and OP, in breast malignant tumor diseases, but also combined multiple bioinformatics methods, which greatly improved the efficiency of toxicological screening. This has significantly enhanced the applicability of network toxicology in the investigation of the pathogenesis of environmental toxins. Under the current research framework of “network toxicology - bioinformatics - molecular docking”, molecular dynamics simulation is a key link connecting “virtual screening” and “experimental verification”, providing real and quantifiable academic evidence for the mechanism study of breast malignant tumors induced by BPA, NP, and OP through multiple dimensions. However, our research has several drawbacks. We neglected to explore direct experimental evidence of exposure to phenolic EDCs in animal models of breast cancer, and large-scale, long-term epidemiological investigations are scarce. Experimental verification is crucial for establishing the trustworthiness of the finding. Future studies should involve experimental validation in both in vitro and in vivo models of breast cancer to verify the primary targets and signaling pathways highlighted by network toxicology. This study has built a robust theoretical platform for further exploring the possible induction of breast tumors and other health concerns connected to environmental pollutants.

## Conclusion

Under the in vitro conditions of this study, by integrating network toxicology, machine learning, multi-omics immune infiltration, molecular docking, and molecular dynamics simulation techniques, it is suggested that phenolic EDCs such as BPA, NP, and OP may be involved in breast cancer-related pathways, providing preliminary clues to the potential risk of promoting breast cancer. It is not yet sufficient to make causal or mechanistic inferences concerning the formation of breast cancer in vivo. Further validation is needed in the future through animal models and population investigations. Through cross-mapping of CTD, SEA, and Swiss Target Prediction, 156 potential targets shared by three chemicals and highly linked with breast cancer were identified for the first time, providing a repeatable resource library for the “multi-contamination-single sickness” study framework. GO/KEGG enrichment suggests that these targets are concentrated in GPCR signaling, JNK cascade, sphingolipids, and prolactin pathways, and are significantly enriched in the “endocrine resistance” and “cell growth and death” modules, which functionally explain the molecular basis of environmental estrogen interfering with breast epithelial homeostasis. The combined feature screening of LASSO and SVM-RFE based on GSE42568 discovered six important genes, including MAOA, MGLL, ADRA2A, RPN2, GF1R, and CTSD. Among them, MAOA, MGLL, and ADRA2A were significantly expressed in cancer tissues, while RPN2, GF1R, and CTSD were noticeably low expressed. Among them, the AUC of MAOA, MGLL, ADRA2A, and RPN2 is ≥ 0.91, suggesting both mechanistic and diagnostic transformation potential. The single-gene GSEA found that the six core genes jointly activated the Wnt, TGF-β, chemokine, and riboflavin metabolic pathways, suggesting that they drive the progression of breast cancer by altering the tumor microenvironment, RNA splicing, and amino acid metabolism. Additionally, ssGSEA immunoassay revealed that the expression of core genes was positively correlated with the infiltration of M2 macrophages, memory B cells, and resting dendritic cells, and negatively correlated with activated CD4 + T cells and activated dendritic cells, providing that environmental endocrine disruptors can promote tumor immune escape by reshaping the immune microenvironment. Molecular docking and molecular dynamics simulations found that BPA has A high affinity for MGLL at −8.7 kcal mol⁻¹, with RMSD ≈ 1.6 A, hydrogen bond number ≈ 3, and RMSF < 2.3 Å. The complex has excellent stability, providing atomic-level evidence for subsequent studies on structure-toxicity relationships. It has been established that the stable binding and interaction between these phenolic metabolites and their targets efficiently bridge the gap between virtual screening and biological reality. Although this multi-dimensional framework has substantially boosted the efficiency of toxicological screening and provided a solid theoretical base, it still has several drawbacks, such as the lack of direct in vivo experimental evidence and long-term epidemiological data. Future research should prioritize experimental validation in both in vitro and in vivo breast cancer models to corroborate the identified primary targets and signaling pathways. Overall, this study has encouraged the application of network toxicology in environmental health and provided new insights into the health concerns posed by phenolic environmental contaminants in breast cancer.

## Supplementary Information

Below is the link to the electronic supplementary material.

Supplementary Material 1

## Data Availability

The dataset generated and analyzed in this study has not been made public as it is still in the incubation stage of the results, but it can be provided by the corresponding author upon reasonable request.
