# Supplementary material for: The contribution of phenolic endocrine-disrupting chemicals to breast cancer risk: A comprehensive bioinformatics analysis
Source: Sci Rep. 2026 Feb 11;16:8283. doi: 10.1038/s41598-026-39706-x (PMC12966292; doi:10.1038/s41598-026-39706-x)
Supplement: Supplementary file 1 — Supplementary Material 1 [file 41598_2026_39706_MOESM1_ESM.docx]

**Supporting Information**

**Molecular Docking Results – Further Explanations:**


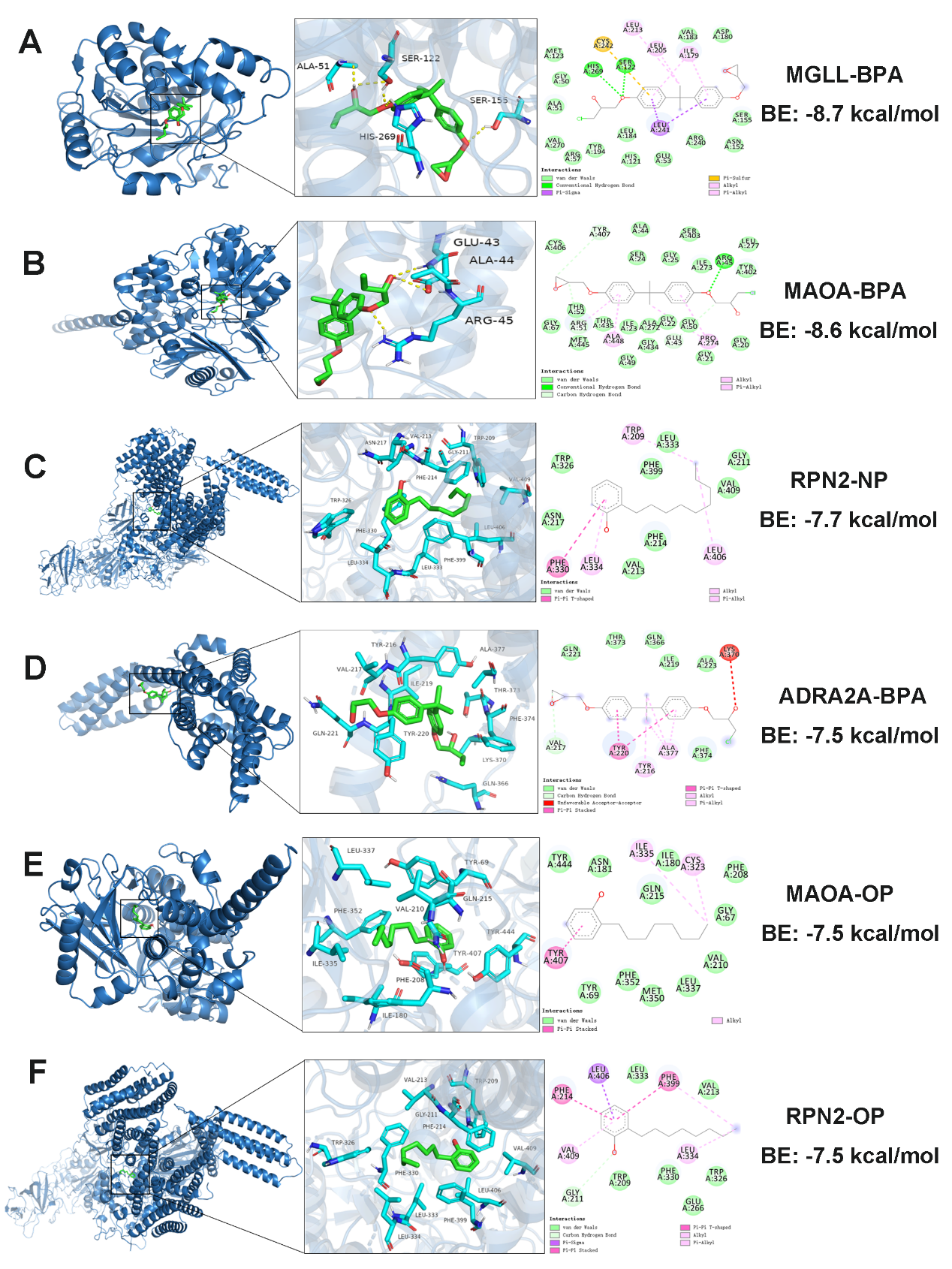


**Fig. 6. Molecular docking results demonstrate the binding affinity of the compound to each core target protein.**

A displays the 3D and 2D visualizable molecular docking outcomes of MGLL and BPA.

The SER122 and HIS269 residues on the MGLL receptor form hydrogen bond interactions with BisphenolA, while the LEU213, LEU205 and ILE179 residues on the MGLL receptor form with BisphenolA Hydrophobic interaction forces: The residues ASP180, VAL183, MET123, GLY50, ALA51, VAL270, ARG57, LEU184, HIS121, GLU53, ARG240, ASN152, and SER155 on the MGLL receptor form van der Waals with Bisphena A Interaction forces: The CYS242 residue on the MGLL receptor forms a Pi-Sulfur interaction force with BisphenolA, and the LEU241 residue on the MGLL receptor forms a Pi-Sigma interaction force with BisphenolA.

B exhibits the 3D and 2D visualizable molecular docking results of MAOA and BPA.

The ARG45 residues on the MAOA receptor form hydrogen bond interactions with BisphenolA, and the TYR407, ARG51, and GLU43 residues on the MAOA receptor form Carbon hydrogen with BisphenolA The bond force is formed by the ALA448, PRO274 and ARG51 residues on the MAOA receptor and BisphenolA Hydrophobic interaction forces, TYR402, LEU277, ILE273, SER403, GLY25, SER24, ALA44, CYS406, GLY67, THR52, MET445, ILE23, THR435, ALA272, GLY22, GLY50 on MAOA receptors GLY49, GLY434, GLY21 and GLY20 residues form van der Waals interactions with BisphenolA.

C demonstrates the 3D and 2D visualizable molecular docking findings of RPN2 and NP.

The TRP209, LEU406 and LEU334 residues on the RPN2 receptor form with Nonylphenol Hydrophobic interaction forces: VAL409, GLY211, LEU333, PHE399, TRP326, ASN217, VAL213, and PHE214 residues on the RPN2 receptor form van der Waals interaction forces with Nonylphenol, while PHE330 residues on the RPN2 receptor interact with Nonylph enol forms Pi-Pi T-shaped forces.

D showcases the 3D and 2D visualizable molecular docking results of ADRA2A and BPA.

The VAL217 residues on the ADRA2A receptor form a Carbon hydrogen bond interaction with BisphenolA, and the ALA377 and TYR216 residues on the ADRA2A receptor form with BisphenolA Hydrophobic interaction forces: The PHE374, ALA223, ILE219, GLN366, THR373, and GLN221 residues on the ADRA2A receptor form van der Waals interactions with Bisphena A, while the TYR220 residue on the ADRA2A receptor forms Pi-Pi with Isphena A The TYR220 residue on the ADRA2A receptor forms a Pi-Pi Stacked interaction with Bisphena A.

E illustrates the 3D and 2D visualizable molecular docking outcomes of MAOA and OP.

The CYS323 and ILE335 residues on the MAOA receptor are formed with Octylphenol Hydrophobic interaction forces: TYR444, ASN181, GLN215, ILE180, PHE208, GLY67, VAL210, LEU337, MET350, PHE352, and TYR69 residues on the MAOA receptor form van der Waals interaction forces with Nonylphenol, and the MAOA receptor The TYR407 residues on the body form Pi-Pi Stacked forces with Octylphenol.

F presents the 3D and 2D visualizable molecular docking results of RPN2 and OP.

The VAL409, LEU334 and PHE399 residues on the RPN2 receptor are formed with Octylphenol Hydrophobic interaction forces: VAL213, LEU333, TRP209, PHE330, GLY266, and TRP326 residues on the RPN2 receptor form van der Waals interaction forces with Nonylphenol, while LEU406 residues on the RPN2 receptor form Pi with Octylphenol The -Sigma interaction force: The PHE214 residue on the RPN2 receptor forms a Pi-Pi Stacked interaction force with Octylphenol, and the PHE399 residue on the RPN2 receptor forms a Pi-Pi T-shaped interaction force with Octylphenol.
